# The Role of Genomics in the Identification, Prediction, and Prevention of Biological Threats

**DOI:** 10.1371/journal.pbio.1000217

**Published:** 2009-10-26

**Authors:** W. Florian Fricke, David A. Rasko, Jacques Ravel

**Affiliations:** Institute for Genome Sciences (IGS), University of Maryland School of Medicine, Baltimore, Maryland, United States of America

## Abstract

In all likelihood, it is only a matter of time before our public health system will face a major biological threat, whether intentionally dispersed or originating from a known or newly emerging infectious disease. It is necessary not only to increase our reactive “biodefense,” but also to be proactive and increase our preparedness. To achieve this goal, it is essential that the scientific and public health communities fully embrace the genomic revolution, and that novel bioinformatic and computing tools necessary to make great strides in our understanding of these novel and emerging threats be developed. Genomics has graduated from a specialized field of science to a research tool that soon will be routine in research laboratories and clinical settings. Because the technology is becoming more affordable, genomics can and should be used proactively to build our preparedness and responsiveness to biological threats. All pieces, including major continued funding, advances in next-generation sequencing technologies, bioinformatics infrastructures, and open access to data and metadata, are being set in place for genomics to play a central role in our public health system.

Since the publication in 1995 of the first complete genome sequence of a free-living organism, the bacterium *Haemophilus influenzae* [1], more than 1,000 genomes of species from all three domains of life—Bacteria, Archaea, and Eukarya—have been completed and a staggering 4,300 are in progress (not including an even larger number of viral genome projects) (GOLD, Genomes Online Database v. 2.0; http://www.genomesonline.org/gold.cgi, as of August 2009). Whole-genome shotgun sequencing remains the standard in biomedical, biotechnological, environmental, agricultural, and evolutionary genomics (http://genomesonline.org/gold_statistics.htm#aname). While next-generation sequencing technology is changing the field, this approach will continue to be used and lead to a previously unimaginable number of genome sequences, providing opportunities that could not have been thought of a few years ago. These opportunities include studying genomes in real-time to understand the evolution of known pathogens and predict the emergence of new infectious agents (Box 1). With the introduction of next-generation sequencing platforms, cost has decreased dramatically, resulting in genomics no longer being an independent discipline, but becoming a tool routinely used in laboratories around the world to address scientific questions. This global sequencing effort has been focusing primarily on pathogenic organisms, which today are still the subject of the majority of genome projects [2]. Sequencing two to five strains of the same pathogen has, in recent years, afforded us not only a better understanding of evolution, virulence, and biology in general [3], but, taken to the next level (hundreds or thousands of strains) it will enable even more accurate diagnostics to support epidemiological studies, food safety improvements, public health protection, and forensics investigations, among others.

**Box 1. Hot Spots for the Emergence of Infectious Disease**

Can we define “hot spots” of microbial populations where new infectious diseases are more likely to evolve? Human contact with new types of infectious agents precedes the emergence of infectious diseases. Infectious agents can be new in the sense of not having previously infected humans or new in the sense that a combination of preexisting genetic factors (for example, mobile elements or regulatory elements) have reassembled to give rise to an infectious agent with a substantially altered genome. The Ebola virus, which first emerged by infecting humans 1976 in Zaire [21], is an example of the former, whereas the acquisition of antimicrobial resistance by *Acinetobacter baumannii* [22] is an example of the latter. In both cases, a change in the selective pressure on an infectious agent allows its emergence from a specific setting. This selective pressure may be, for example, the new niche that the human host provides to the pathogen or the antimicrobial selection on a pathogen. Since both events rely on preexisting genetic resources and not on the de novo evolution of virulence factors, the potential of a setting to serve as a hot spot or reservoir for an emerging infectious disease is theoretically predictable from the examination of the total metagenome. In this scenario, traditional microbiological approaches that focus on single isolates of bacteria or viruses are limited in their predictive power since they lack a view of the complete genetic landscape. The potential infectious disease agent could, however, arise from an environment that only contains pieces of a “virulence puzzle,” i.e., individual virulence factors encoded within the genomes of different organisms (the metagenomic “gene soup”). These pieces would have to be assembled in one species for the new pathogen to emerge as an infectious agent.

## Biodefense Funding for Genomic Research

Since the anthrax letter attacks of 2001, when letters containing anthrax spores were mailed to several news media offices and two Democratic senators in the United States, killing five people and infecting 17 others, funding agencies in the US and other countries have prioritized research projects on organisms that might potentially challenge our security and economy should they be used as biological weapons. This has resulted in large amounts of funding dedicated to so-called “biodefense” research, totaling close to ░50 billion between 2001 and 2009 [4]. Genomics has benefited greatly from this influx of research dollars and as a result, representatives of most major animal, plant, and human pathogens have been sequenced (http://www.pathogenportal.org/). Supported by federal funds from the National Institutes of Health (NIH), the National Institute of Allergy and Infectious Diseases (NIAID), and the US Department of Defense, research programs, such as the Microbial Sequencing Centers and the Bioinformatics Resource Centers (http://www3.niaid.nih.gov/topics/pathogenGenomics/PDF/genomicsinitiatives.htm), have been established that carry out genomics research on pathogenic organisms and have spearheaded a new phase of the genomics revolution. Similar programs were started in Europe, such as those at the Wellcome Trust Sanger Institute in the United Kingdom, and the multinational European effort, The Network of Excellence EuroPathoGenomics (http://www.noe-epg.uni-wuerzburg.de/epg_general.htm). As an example of the success of these types of programs, the genome sequences of over 90,000 influenza viruses were rapidly generated and are now deposited in GenBank (http://www.ncbi.nlm.nih.gov/genomes/FLU/aboutdatabase.html). Because of the availability of large sequencing capacity and the large amount of information, the response to the 2009 H1N1 influenza pandemic was rapid and efficient (Box 2): Genomics information was generated within days and validated diagnostic tools were approved within weeks [5],[6]. A global response was made possible through tremendous research efforts enabled by genomic research.

**Box 2. Pandemic H1N1 2009 Influenza: A Recent Example of the Impact of Genomics on Biopreparedness**

Genomics can be readily applied to follow outbreaks of infectious diseases. This is clearly illustrated during the severe acute respiratory syndrome (SARS) outbreak in 2002–2003 and the emergence and worldwide spread of the pandemic H1N1 2009 influenza virus this year. In both cases, genomics played a key role in the immediate response to the outbreak. Initially, very little was known about the virus responsible for the SARS outbreak. Pangenomic virus microarrays identified it as a coronavirus [23]; however, it was only through detailed sequencing that the specific genotype of this virus could be determined [24]. Comparative sequence analysis identified the SARS virus as distinct from other coronaviruses in terms of its encoded proteins responsible for antigen presentation. This finding ultimately lead to development of diagnostics [25] and potential therapeutics [26]. This example of a sequencing approach as a rapid response to a virus outbreak demonstrates that genomics can be a useful and important, if not essential, epidemiological tool. In the ongoing H1N1 influenza outbreak, the National Center for Biotechnology Information (NCBI) established the Influenza Virus Resource (a database and tool for flu sequence analysis, annotation, and submission to GenBank; http://www.ncbi.nlm.nih.gov/genomes/FLU/SwineFlu.html), containing 462 complete viral genome sequences from worldwide viral samples (as of September, 2009). Some of the genomic data was completed, compared, and released to the public within two weeks of isolation of the DNA. The rapid generation of genome sequence data is providing a paradigm shift in the analysis of infectious disease outbreaks, from more classical methods of isolation to the rapid molecular examination of the pathogen in question.

## Access to and Documentation of Sequence Data

Open access to genomics resources (i.e., raw sequence data and associated publications) is an essential component of the nation preparedness to biological threats (biopreparedness), whether intentionally delivered or not. Although some consider open-source genomic resources a threat to security [7] because they make publicly available information that could facilitate the construction of dangerous infectious agents, we strongly disagree with this point of view. Rather, we and others [8] believe that it is an enabling tool more useful to those in charge of our public health and biosecurity than to those with ill intentions. Genomic sequence data can provide a starting point for the development of new vaccines, drugs, and diagnostic tests [9], hence improving public health capabilities and increasing our biopreparedness. Access to the organisms from which the sequences are derived should be restricted, not their genome sequences. Now that genomics technologies are broadly available, there is the potential for commercial interests to hamper the release of genomic data in the public domain. Thus it is important that federally funded large-scale genome sequencing efforts have enforceable rapid release policies. This accessibility could afford further opportunities to capitalize on investments in genome sequencing by providing the necessary resources to biopreparedness.

Whereas genome projects aimed at sequencing one, two, or three isolates of a pathogen seemed adequate a few years ago, it is now possible to sequence rapidly hundreds of individual genomes for each species. Access to relevant, well-curated culture collections [10] and DNA preparations suitable for sequencing may become a bottleneck in the future when sequencing resources are no longer limiting. More importantly, the impact of large genomic sequence datasets from clinical isolates will be limited without key clinical metadata that characterize these isolates, such as patients' medical information, date of isolation, and the number of culture passages in the laboratory. Open access to large numbers of sequences and associated metadata allows for powerful comparative genomic analyses and thus provides major insights into the characteristics of a pathogen. Standardized vocabulary should be developed to describe these isolates and the genes they contain. Such efforts have already started, for example through the open-access journal Standards in Genome Sciences (SIGS) (http://standardsingenomics.org/index.php/sigen), but the dedicated resources are not adequate and highlight the lack of understanding of the importance of metadata in genomics. Initiatives such as those of the Genomics Standards Consortium have made great strides [11],[12], but still need widespread implementation from the ever-expanding genomic community. Open access to the genomic DNA that has been sequenced or the culture from which the DNA was extracted and to the associated metadata is key to successful genome sequencing projects, whether on single or several hundred genomes or metagenomes. Well-documented genome sequence data will form a key growing resource for biodefense and other research fields.

## Emerging New Bioinformatics Resources

As we enter a new era of modern genomics, the ever-expanding sequence datasets are becoming more challenging to analyze. Future analysts will require powerful new bioinformatics tools in conjunction with new computer systems engineered with genomic analysis in mind. Open-source new bioinformatics software tools are being developed that exploit Web-based services and the increasing computing power provided by academic and commercial “cloud computing networks” (large computing resources provided as a service over the Internet). For example, “Science Clouds” (http://workspace.globus.org/clouds/) allow members of the scientific community to lease cloud computing resources free of charge. To leverage these capabilities, novel cloud-optimized bioinformatics tools are being developed, such as the genome sequence read mapper CloudBurst [13]. In addition, novel resources are currently under development to increase the availability of open-source bioinformatics tools for cloud computing (http://www.nsf.gov/awardsearch/showAward.do?AwardNumber=0949201; http://www.nsf.gov/awardsearch/showAward.do?AwardNumber=0844494). These emerging tools make access to the Worldwide Web the only requirement to join the genomic revolution and achieve large scale bioinformatics analyses that could not be possible on local servers. As a consequence, it is conceivable that in the future genomic research will increasingly move away from the large sequencing centers toward a more decentralized organization. Decentralized rapid genome sequencing and bioinformatic analysis of infectious agents will enable near-real-time global surveillance, detection of new pathogens, new virulence factors, antimicrobial resistance determinants, or engineered organisms.

## Population Genomics Applied to Single Cultures

Because the resources for affordable high-throughput sequencing, data processing, and analysis are available, the time is right to think about microbial population genomics and large-scale microbial metagenomics in the context of biodefense research (Box 3). Traditionally, the concept of population genomics has applied to variation within a species. However, a bacterial culture, even if derived from a single clone, is composed of millions of cells that are not necessarily identical at the genome sequence level, hence forming a population of genomes. Therefore we propose to apply the concept of population genomics to microbial cultures. The assemblage of genotypes defines what is called a “culture,” “culture stock,” or “reference strain.” Population genomics addresses the genomic diversity within these assemblages and has significant implications for many fields of research but, most importantly, for pathogen evolution, diagnostics, epidemiology, and microbial forensics. For example, following the anthrax mail attacks of 2001, microbiologists and genomicists joined forces to characterize the unique genetic traits of the *Bacillus anthracis* spores recovered from the envelopes, which were quickly identified as the *B. anthracis* Ames strain (DAAR et al., unpublished data). Sequencing the genome of several single colonies obtained from the spores revealed that the entire chromosome and its associated plasmids were 100% identical to the genome sequence of the ancestral *B. anthracis* Ames strain that was stored for over 20 years in a military laboratory in Frederick, Maryland. The only genotypic differences were found in a small, phenotypically and genetically distinct portion of cells grown from the spores used in the attacks. Genomic characterization of these phenotypic variants revealed a number of unique genetic alterations that together provided a characteristic DNA fingerprint of the spore population that could be unequivocally matched to the spore sample used in the attacks. Using this fingerprint, a genetic assay was developed to screen a *B. anthracis* spore repository, which identified the origin of the spores as a single spore stock of *B. anthracis* Ames. This stock was stored at the US Army Medical Research Institute for Infectious Diseases in Fort Detrick, Maryland, narrowing the pool of suspects to a manageable number (those who had access to the spore stock) for the investigative team. The police investigation that followed identified a potential suspect as the custodian of the spore stock. This was the first use of microbial genomics as an essential tool in a forensic investigation. In the course of the investigation, scientists had to establish culture repositories from strains used in research in the US and build databases of genome sequences of all *B. anthracis* isolates. This work took several years and delayed the investigation significantly. A lesson to be learned from this investigation should therefore be that there is a need for comprehensive databases of unique DNA fingerprints of stocks of potentially threatening pathogens. In the event that another bioterror attack were to take place such genomic databases would be key in quickly establishing the source of the biological material.

**Box 3. Simple Genomics, Population Genomics, and Metagenomics**

It is now technically possible and scientifically desirable to combine sequencing projects on single genomes, genome populations, and metagenomes to study genome evolution. Single-genome projects provide the greatest resolution for identifying genetic factors responsible for specific virulence phenotypes and provide answers to many important questions, such as: What is the minimal gene set in a pathogen required to cause a specific disease phenotype? What does the genetic context of virulence or antibiotic resistance factors tell us about their evolutionary origin or the mobility between different microbial species or even genera? Population-level genome sequencing projects provide us with information about the pangenomic gene pool and the potential of a species to evolve into a novel pathogen. Are certain bacterial species or strains more likely than others to evolve pathogenic traits? What distinguishes a commensal from a pathogenic isolate? What provides the trigger or ability to convert a commensal or opportunistic strain into a pathogen? What role does horizontal gene transfer play in species evolution? Is an infection always caused by an individual isolate or might infection be caused by a combination of individuals in a population that all have different attenuated infectious potentials? Metagenomics projects sample the genetic reservoir (the set of genes carried by all members of a community) within a specific environment or sample. This “gene soup” reflects the maximum genetic potential accessible to individual isolates by horizontal gene transfer.

The concept of population genomics also applies to epidemiological studies of outbreaks of infectious diseases such as those caused by food-borne or zoonotic pathogens, such as *Salmonella* spp. Traditionally, epidemiologists and pathologists have used low-resolution methods such as pulsed-field gel electrophoresis (PFGE), multi-locus sequence typing (MLST), or multi-locus variable number tandem repeats analysis (MLVA) to trace an individual isolate from a patient back to a potentially infected food source or to isolates from other patients [14]–[17]. In 2006, for example, during an outbreak of pathogenic *Escherichia coli* O157:H7 infections in 26 states of the US, which was caused by contaminated spinach, isolates of the pathogen were recovered from cows and wild pigs (the zoonotic reservoirs), bags of spinach (the vehicle of transmission), and ill patients (http://www.cdc.gov/mmwr/preview/mmwrhtml/mm55d926a1.htm). One of these isolates was designated as the reference for the outbreak based on conserved PFGE patterns. Genome sequencing of several isolates from the same outbreak performed in our laboratory, however, revealed genomic variations that questioned a direct evolutionary link between all outbreak-associated isolates (Eppinger et al., unpublished data). Comparative genomics followed by whole-genome phylogenetic analyses based on single nucleotide polymorphisms demonstrated that these isolates were indeed closely related to one another and only distantly related to other *E. coli* O157:H7 isolates, hence linking all isolates to the same outbreak, something that was not possible using PFGE patterns. In this case, phylogenetic analyses suggest that several highly related genotypes were at the source of the outbreak, thus challenging the utility of assigning a single reference strain to a specific outbreak. Instead, collecting and sequencing tens or hundreds of isolates from each source or patient linked to an outbreak would provide a better basis for understanding the genomic diversity within the outbreak population and would aid in defining the population dynamics of an outbreak.

## A New Concept: Contrabiotics

Insufficient attention has been paid to the human microbiome (i.e., the consortium of microbes that inhabit the human body) as it relates to our efforts to increase biopreparedness. New analyses of the diversity and composition of the human microbiome are making it increasingly clear that human health depends on a delicate equilibrium between the microbial inhabitants and the human host [18],[19]. Severe effects on health could be caused not only by the introduction of true pathogens in the traditional sense into these human-associated microbial communities (e.g., *Vibrio cholerae*, the etiologic agent of cholera) but potentially also by slight shifts in the proportions of different populations within the community that give an otherwise harmless species or strain an undesirable advantage over others, a similar situation to what is observed in bacterial vaginosis [20]. Probiotic dietary supplements of live microorganisms deliver beneficial bacteria that promote an healthy state of the targeted microbiota. In a completely hypothetical possibility, the opposite would also be plausible, where the healthy microbiota (skin, gut, or upper respiratory tract, among others) may be disturbed by introducing large amounts of “contrabiotics,” i.e., living nonpathogenic bacteria that would shift the microbiota away from a healthy state. A better understanding of the ecological principles that shape the composition of our microbiome might contribute to our biopreparedness for such a threat to public health.

## Challenges for the Future

The field of biodefense has thoroughly embraced genomics and made it a keystone for developing better identification technologies, diagnostic tools, and vaccines and improving our understanding of pathogen virulence and evolution. Enabling technologies and bioinformatics tools have shifted genomics from a separate research discipline to a tool so powerful that it can provide novel insights that were not imaginable a few years ago, including for example redefining the notion of strains or cultures in the context of biopreparedness or microbial forensics. Challenges remain, though, mostly in the form of large amounts of data that are being generated, and will continue to be generated in the future, and are becoming difficult to manage. The need for better bioinformatic algorithms, access to faster computing capabilities, larger or novel and more efficient data storage devices, and better training in genomics are all in critical demand, and will be required to fully embrace the genomic revolution. Our nation's preparedness for biological threats, whether they are deliberate or not, and our public health system would benefit greatly by leveraging these capabilities into better real-time diagnostics (in the environment as well as at the bedside), vaccines, a greater understanding of the evolutionary process that makes a friendly microbe become a pathogen (Box 3) (hence to better predict what microbial foes will be facing us in the near future), and better forensics and epidemiological tools. The time is right to be bold and capitalize on these enabling technological advances to sequence microbial species or complex microbial communities to the greatest level possible—that is, hundreds of genomes per species or samples—but let us not forget that informatics and computing resources are now becoming the bottleneck to actually making major progress in this field.
